# Cardiac Biomarkers in Patients with Acute Pulmonary Embolism

**DOI:** 10.3390/medicina58040541

**Published:** 2022-04-14

**Authors:** Luc Janisset, Maxime Castan, Géraldine Poenou, Raphael Lachand, Patrick Mismetti, Alain Viallon, Laurent Bertoletti

**Affiliations:** 1 Service des Urgences, CHU de St-Etienne, F-42055 Saint-Etienne, France; luc.janisset@chu-st-etienne.fr (L.J.); maxime.castan@icloud.com (M.C.); alain.viallon@chu-st-etienne.fr (A.V.); 2 INSERM, UMR1059, Equipe Dysfonction Vasculaire et Hémostase, Université Jean-Monnet, F-42055 Saint-Etienne, France; patrick.mismetti@chu-st-etienne.fr; 3 Service de Médecine Vasculaire et Thérapeutique, CHU de St-Etienne, F-42055 Saint-Etienne, France; geraldine.poenou@chu-st-etienne.fr; 4 Service de Médecine Intensive et Réanimation, CHU de St-Etienne, F-42055 Saint-Etienne, France; raphael.lachand@chu-st-etienne.fr; 5 INSERM, CIC-1408, CHU Saint-Etienne, F-42055 Saint-Etienne, France

**Keywords:** pulmonary embolism, cardiac biomarker, troponin, NT-proBNP, sPESI

## Abstract

Pulmonary embolism is a frequent and potentially fatal disease. The major challenge of initial management lies in prognostic stratification. Since 2014, the European recommendations on the diagnosis and management of acute pulmonary embolism are based on assessing the risk stratification regarding hemodynamic status first, then on a combined risk assessment model using a clinical score, an imaging evaluation of right heart size and the concentration of a serum cardiac biomarker. Usual biomarkers cover cardiac ischemia (troponin and derivates) and dilatation (BNP and derivates). The aim of this review is to offer a practical update on the role of the Troponins and BNPs families of biomarkers and the prognosis of pulmonary embolism, and furthermore, to provide a brief overview of their place in current management.

## 1. Introduction

Pulmonary embolism (PE) is a frequent and potentially fatal disease [1,2]. Its annual incidence is estimated to be between 60 and 80 cases/100,000 inhabitants in Europe [3]. Despite the optimization of management, overall mortality remains high, between 3 and 12% 30 days after the diagnosis and up to 50% in PE patients with categorized at “high-risk” of adverse events in the first month after the diagnosis [1,2]. The major challenge of managing this pathology firstly lies in rapid assessment of an accurate prognostic stratification. Since 2014, the European recommendations [4] on the diagnosis and management of acute pulmonary embolism are based on assessing the risk stratification regarding hemodynamic status. Indeed, diagnosis of hemodynamic instability leads to immediate introduction of un-fractioned heparin with discussion of thrombolysis. In the absence of hemodynamic instability, prognostic stratification assessment is based on a combined risk assessment model using a clinical score, an imaging evaluation of right heart size and the concentration of a serum cardiac biomarker. For the hemodynamically stable patient, the clinical state is evaluated using the HESTIA rule, the Pulmonary Embolism Severity Index (PESI) [5], or its simplified version the sPESI [6]. The sPESI is the most commonly used risk assessment model and classifies patients into three risk levels; low, intermediate and high risk patients [1,2]. In the low risk category, the assessment relies only on clinical parameters. In the high risk category, medical urgency prevails over any type of complementary exploration. For intermediate risk patients, the clinical risk stratification is combined with the assessment of the right heart cavity using echocardiography or CT scan and serum cardiac biomarkers. An echocardiography-derived or a CT-derived right-to-left ventricular dimension ratio (RV/LV ratio) over one predicts a higher risk of an upcoming adverse event. Similarly, an increase in serum biomarkers of myocardial ischemia (such as Troponin I or Troponin T) or myocardial stretch (such as NT-proBNP or proNBNP) indicates the failure of the adaptation mechanisms of the right ventricle and suggests an increased risk of hemodynamic instability. Troponins and natriuretic peptides are not specific to VTE and can increase in various pathological situations, yet have the advantage of being more accessible than echocardiography or CT scan. Therefore, a careful understanding of their meaning in pulmonary embolism patients is needed. The aim of this review is to offer a practical update on the role of the Troponins and BNPs families of biomarkers and the prognosis of pulmonary embolism and to provide a brief overview of their place in current management. 

## 2. The Place of Troponins in the Management of Patients with PE?

Troponin is a protein complex of the cytoskeleton of muscular cells in addition to actin filaments, microtubule and myosin filaments. The Troponin complex is composed of three subunits, Troponins I, T and C [7]. Each of these subunits has a specific role in the contraction of myocytes. Troponin I prevents myosin from binding to actin in relaxed muscle. Troponin T binds to tropomyosin, forming the troponin-tropomyosin complex. Troponin C binds the Calcium necessary for the complex activation. Troponin complex activation causes changes of the complex shape exposing binding sites for myosin on the actin filaments allowing muscle contraction. Cardiac subtype of troponin I and T are specific predictors of the efficiency of the contraction of cardiac myocytes [8] and Troponin T serum level is the most sensitive and specific in measuring cardiac damage. Based on data from studies on acute myocardial infarction, cardiac Troponins need 4–10 h to appear in serum after the event and peak after 12–48 h Therefore, Troponin T is mainly used as a early marker in the strategy of acute myocardial infarction. The normal serum level of Troponin T is inferior to 14 ng/L. However, any clinical situations involving a difficulty in contractility of the myocyte will induce a troponin complex destruction and an increase in troponin T serum level, like pulmonary embolism (Table 1). Troponins are cleared by the kidney and remain abnormal for several days [9]. In pulmonary embolism, parietal stress related to the rapid increase in right ventricle afterload leads to a Troponins release probably related to micro-ischemia, as a result of wall tension [10]. An elevation of this marker is noted in coronary syndrome or pulmonary embolism, even if the elevation in the latter situation seems less important. An isolated Troponin elevation does not indicate whether the right or left ventricle is affected [11]. However, combined with an electrocardiogram, the side of the cardiac damage left or right can be specified. 

Troponins were shown to be markers of the risk of complications 30 days after pulmonary embolism [12,13,14]; initially Troponin I, then hyper sensitive Troponin T (hsTnT) alone [15]. Afterwards, it was demonstrated that combining Troponin results with a clinical score (PESI or its simplified version, sPESI) improved prognostic stratification of patients with PE [16]. 

At any rate, abnormally elevated Troponin levels are associated with an increased risk of mortality in unselected PE patients (OR 5.2, 95% CI 3.3–8.4), and also when only hemodynamically stable patients were analyzed (OR 5.9, 95% CI 2.7–13.0) [17].

The assessment of Troponin levels is currently primarily used for their reliable negative predictive value, and thus, mainly for the separation of a subgroup of PE patients with low risk of degradation, making them eligible for outpatient management [1,2]. 

## 3. The Place of BNP in the Management of Patients with PE 

Cardiac natriuretic peptides include atrial natriuretic peptide (ANP) and B-type natriuretic peptide (BNP). BNP is the second biomarker used in the stratification risk of an adverse event in acute PE patients [18]. The exact pathophysiological trigger of production and secretion of BNP remains unclear, however, BNP plays a role in sodium homeostasis and blood regulation. Therefore, increased levels of BNP lead to systemic vascular and pulmonary arterial relaxation. Pro-BNP is cleaved into BNP and N Terminal proBNP (NT pro BNP), which has no physiological activity yet has a plasma concentration five to ten times higher than BNP, making it more suitable for measuring. BNP half-life is shorter than NT Pro BNP (20 min versus 2 h) BNP and NT pro BNP are cleared by the kidney. In general, the plasma concentrations of these peptides are increased in diseases characterized by ventricular hypertrophy or strain (Table 1) [19]. Several elements interfere in the interpretation of the normality of a BNP or NT Pro BNP level. Reference values are typically higher in women and elderly patients, although only age should modify the thresholds used. Kidney failure has a high impact on the level of NT Pro BNP [20].

The increase in RV afterload induces an RV dilatation, which causes the release of BNP and its precursor NT-proBNP in patients with PE [1,2]. In 2003 the first study was published [21] with an abnormal level of NT-proBNP being associated with an increased risk of PE-specific death or an adverse outcome. Subsequently, meta-analyses confirmed in an unselected patient population with PE that patients with abnormal levels of BNP or NT-proBNP were six to seven times more likely to die or to present with adverse clinical outcome [22]. Importantly, half of the patients included in the meta-analysis had an abnormal cardiac biomarker, highlighting the clinical relevance of the biomarker. In these patients, the risk of early death was as high as 10% [95% CI 8.0–13%], while the risk of adverse events like admission to the intensive care unit, any use of resuscitation treatment or more invasive PE treatment was 23% [95% CI 20–26%]. In a study published in 2010 [23], the accuracy of NT-pro-BNP for identification of PE patients with a low risk of adverse events was superior to the prediction associated with imaging derived RV/LV ratios.

The assessments of BNP and NT-proBNP markers levels are currently used for their good negative predictive value, due mainly for the individualization of a subgroup of PE patients with low risk of degradation, eligible for outpatient management [1,2].

## 4. The Place of the Potential Other Biomarkers

Three markers are currently discussed to be integrated into the risk assessment of adverse events in the 30 days after the diagnosis of PE: Copeptin, Heart-type fatty acid binding protein and D dimer. 

### 4.1. Copeptin

When cardiac input decreases or during cardiac stress, the hypothalamus secretes arginine vasopressin (AVP). Copeptin (CT-proAVP)) is the C-terminal part of the prevasopressin protein the precursor of AVP. The normal serum level of CT-pro-AVP range is 1.70–11.25 pmol/L. The half-life of CT-proAVP permits an easier testing than AVP and is therefore a reliable AVP surrogate. CT-proAVP has been identified as a reliable biomarker for diagnosis and risk stratification in numerous cardiovascular conditions and also for PE patients, in whom it independently stratified patients at intermediate risk of adverse event in the month following the diagnosis of pulmonary embolism [24]. (Table 1) In PE, the threshold value of CT-proAVP has been established at 24 pmol/L, coinciding with a 6.3-fold increase in the risk of an adverse event and a 7.6-fold increase in the risk of death from pulmonary embolism, in an external prospective validation study [25]. CT-proAVP fast release kinetics allows for prognostic assessment of acute diseases, e.g., in the emergency department. However, actual assays to measure CT-proAVP can take up to 2.5 h before the results are available, while measurements for Troponin and BNP only take 5 min with point of care devices. Moreover, CT-proAVPlevels are increased in patients with diabetes mellitus or kidney failure, like BNPs or Troponins. Currently CT-proAVP is not recommended in risk assessment in daily practice.

### 4.2. Heart-Type Fatty Acid Binding Protein

Heart-type fatty acid binding protein (H-FABP) is a small (15 kDa) myocardial protein used as a biomarker of myocardial ischemia. H-FABP is a protein involved in the fatty metabolism. H-FABP is detected in the blood 1–3 h after the onset of a chest pain during myocardial infarction. Even if H-FABP has good predictive value, it is recommended to be measured simultaneously with troponin. H-FABP has better performance in predicting an adverse outcome in the first month following the diagnosis and long-term mortality, both in hemodynamically stable and instable patients with PE [26] and therefore could be a useful marker in risk stratification too [27]. However, the lack of accessibility in routine and standardization of the measurement assay remain the major limitation [28]. Furthermore, even if it is quickly released into the circulatory system it is then eliminated by the kidney like the other factors and therefore exposed to the same confounding factor [29]. Currently H-FABP is not recommended in risk assessment in daily practice.

### 4.3. D-Dimer

Finally, D-dimer, in addition to being a diagnostic marker when associated to a clinical prediction rule, predicts disease severity but not long-term prognosis in acute PE [30]. 

## 5. What Are the Unmet Needs Using Cardiac Biomarkers?

As a reminder, Troponin and BNP levels are useful to assess the risk of intermediate short-term risk of adverse events in patients with acute PE. To answer this question of unmet needs, it is important to establish the scope of the use of these serum biomarkers, to define to which extend it can be useful in other risk situations, and to determine if the need that they are supposed to meet is clearly being met. 

### 5.1. Is There an Impact of Abnormal Troponin and BNP Levels in PE Patients Clinically Assessed at Low-Risk of Death?

**Figure 1:**
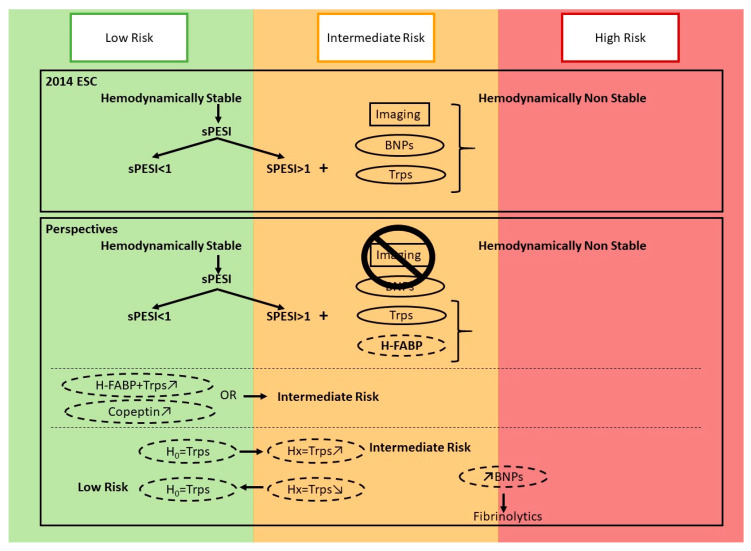
The new potential role of the cardiac biomarkers in acute PE.

According to current guidelines, RV evaluation is not mandatory in patients evaluated as low-risk of short-term mortality, for example those with a null sPESI [1,2]. However, PE diagnosis is usually obtained after blood tests (which may include cardiac biomarkers for alternative diagnosis) and/or chest imaging. Both tests may indicate abnormal RV function, however, how these findings impact the prognostic stratification of patients is unclear. A recently published individual patient data meta-analysis reported that the abnormal level of both Troponins and natriuretic peptids was associated with an increased risk of death at day 30, even in patients assessed as being at low risk of death within clinical models (PESI, sPESI or Hestia) [31] (Table 2). If these results are integrated in the next PE guidelines [32], clinicians will be able to identify patients with a pulmonary embolism with a low risk of death (according to sPESI) and RV dysfunction either with the scanner or with the biomarkers at the same time. Figure 1 summarizes the potential place of each biomarker in the risk assessment of acute PE 30 days after the diagnosis.

### 5.2. What Is the Evolution of Cardiac Biomarker Serum Concentration in Patients with Acute PE?

As described before, it has been established that in acute myocardial infarction troponin measurements should be repeated several times, yet little is known about the clinical significance of biomarkers’ level variations over time in acute PE patients. To our knowledge, there is one study that has reported the concentration variation in Troponin, in the days following hospital admission for pulmonary embolism [34]. 

In a prospective single-center study including 200 patients treated for hemodynamically stable pulmonary embolism, in a similar fashion to Troponin assessment/cycling in acute coronary syndromes, Troponin measurement was repeatedly performed at admission, and then every 8 h for 72 h. Authors demonstrated a misclassification/an improper risk stratification of 30% of patients with initial negative troponin that became positive at the second evaluation, 8 h after admission. Given the variability of symptoms’ duration before hospital admission (from less than 24 h to more than 3 weeks), larger studies are needed to confirm these data.

### 5.3. Will Biomarkers Allow Individualization of a Population, in Which There Is an Increased Risk of Initial or Longer-Term Deterioration?

Briefly after PE, the main issue is to die from fatal PE, while the main long-term issue is to develop chronic thromboembolic pulmonary hypertension (CTEPH). 

In patients stratified at intermediate risk of death in the 30 days after PE, the different options (inferior vena cava filter, thrombolytic, for example) assessed to improve the prognosis did not succeed. In patients with a risk factor for fatal PE, insertion of a temporary inferior vena cava filter was not associated with an improved survival [35]. In patients with PE, RV dilatation and an abnormal level of Troponin, fibrinolytics treatment decreased the risk of death or hemodynamic decompensation, but at the cost of an increased risk of major bleeding [36]. Hence, some authors suggested that higher cut-off values of NT-proBNP may help to better individualize patients at higher risk of degradation [37] and in whom advanced therapy (like thrombolytics) may be justified. 

After PE, RV dysfunction may persist in about one in three patients. The post-PE syndrome has been recently recognized as a potentially distinct phenotype within patients, in whom Chronic thromboembolic pulmonary hypertension (the most severe long term complication after PE) [38] should be assessed. In the last version of the ESC/ERS guidelines, the use of NT-proBNP as screening tools for Chronic thromboembolic pulmonary hypertension has been proposed, in patients with persistent dyspnea and an abnormal RV showing after transthoracic echocardiogram. Further research is needed to elucidate the place of cardiac biomarkers in the screening of post-PE complications. 

## 6. Conclusions

The prognostic value of a biomarker corresponds to its ability to predict the clinical course of the disease in the absence of treatment and therefore to identify patients at high or low risk of occurrence of an adverse event. In acute PE, biomarkers are taking an increasingly prominent place. Nevertheless, they still need to refine their discriminating skills. To date, they have neither the ability to identify patients at intermediate or high risk in an isolated manner, nor to be used as a surrogate for imaging examinations.

## Figures and Tables

**Table 1:** Clinical situation causing elevation of biomarkers in the serum.

Biomarkers	Most Common Use in Clinical Practice	Others Causes of Elevation
Troponin	Myocardial infarction	Acute rheumatic fever, Amyloidosis, Cardiac trauma, cancer therapy, congestive heart failure, critically ill patients, end-stage renal failure, glycogen storage disease type II, heart transplantation, hemoglobinopathy hypotension or hypertension, hypothyroidism, myocarditis/pericarditis, post-operative non cardiac surgery, pulmonary embolism, sepsis,
Natriuretic peptids	Acute heart failure	Acute and chronic pulmonary pathology with right ventricular repercussions, Valvular diseases, Primary and secondary left ventricular hypertrophy, Renal failure, Atrial arrhythmia Sepsis, Acute myocardial ischemia, Chronic systolic dysfunction, Hyperthyroidism, Cushing’s disease or taking corticosteroids, Primary hyperaldosteronism, Addison’s, diabetes, cirrhosis with ascites, paraneoplastic syndrome, subarachnoid hemorrhage
Arginine vasopressin (AVP) and copeptin (CT-proAVP)	Fluid disorders	Myocardial infarction, Cardiogenic shock, Heart failure and Stroke. Inappropriate antidiuretic hormone secretion, Diabetes, Renal failure
Heart-type fatty acid binding protein	Myocardial infarction	Pulmonary embolism, neurodegenerative disease, end stage kidney failure
D dimers	Venous thrombotic events	Atrial fibrillation, Hepatopathy, Advanced age, Hospitalization, Alzheimer, Chronic Inflammation, Aneurysm, Local or systemic inflammation, Arthritis, Heart failure, Burns, Cancer, Nephropathy, Ischemic heart disease, Pancreatitis, Recent surgery, Neonatal period, Disseminated Intravascular Coagulation (DIC) Post Transplantation, Aortic Dissection Acute Respiratory Distress Syndrome (ARDS), Pregnancy and postpartum, Thrombolysis, Disability Arterial or venous thrombosis, Hemolysis during a sickle cell crisis, Trauma, Hemolysis liver (enzyme) low patelet syndrome, Severe urticarial, Hemorrhage

**Table 2:** Laboratory tests for prediction of early mortality in acute PE.

Biomarker	Cut-Off Value	Sensitivity, % (95% CI)	Specificity, % (95% CI)	NPV, % (95% CI)	PPV, % (95% CI)	OR or HR, % (95% CI)	Study Design
NT pro BNP	≥600 pg/mL	81	56	99	1.9	8..7 (2.8–27)	Meta analysis [22]
BNP	75–100 pg/mL	NR	NR	NR	NR	6.5 (2.0–21)	Meta analysis [22]
Troponin T	≥14 pg/mL for patients <75 years	87	42	98	9	4.97 (1.71–14.43)	Prospective cohort [16]
	≥45 pg/mL for patients ≥75 years	83 (55–95)	64 (58–70)	99	11	9.05 (1.94–42.26)	Prospective cohort [33]
H-FABP	6 ng/mL	71 (for clinical complication course at 30-day)	74 (for clinical complication course)	100	41	17.67 (46.02–51.89)	Meta-analysis [27]
		90 (for mortality at 30-day)	72 (for mortality at 30-day)			32.94 (8.80–123.21)	
Copeptin	≥24 pmol/L	62 (41–79)	80 (77–82)	99	7	6.33 (2.58–15.51)	Prospective cohort [25]

## Data Availability

Not applicable.

## References

[1] Silverstein M.D., Heit J.A., Mohr D.N., Petterson T.M., O’Fallon W.M., Melton L.J. (2014). Trends in the incidence of deep vein thrombosis and pulmonary embolism. Arch. Intern. Med..

[2] Konstantinides S.V., Meyer G., Becattini C., Bueno H., Geersing G.-J., Harjola V.-P., Huisman M.V., Humbert M., Jennings C.S., Jiménez D. (2020). 2019 ESC Guidelines for the diagnosis and management of acute pulmonary embolism developed in collaboration with the European Respiratory Society (ERS). Eur. Heart J..

[3] Delluc A., Tromeur C., Le Ven F., Gouillou M., Paleiron N., Bressollette L., Nonent M., Salaun P.-Y., Lacut K., Leroyer C. (2016). Current incidence of venous thromboembolism and comparison with 1998: A community-based study in Western France. Thromb. Haemost..

[4] Konstantinides S., Torbicki A., Agnelli G., Danchin N., Fitzmaurice D., Galie N., Gibbs J.S.R., Huisman M., Humbert M., Kucher N. (2014). 2014 ESC Guidelines on the diagnosis and management of acute pulmonary embolism. Eur. Heart J..

[5] Aujesky D., Perrier A., Roy P.-M., Stone R.A., Cornuz J., Meyer G., Obrosky D.S., Fine M.J. (2007). Validation of a clinical prognostic model to identify low-risk patients with pulmonary embolism. J. Intern. Med..

[6] Jiménez D., Aujesky D., Moores L., Gómez V., Lobo J.L., Uresandi F., Otero R., Monreal M., Muriel A., Yusen R.D. (2010). Simplification of the pulmonary embolism severity index for prognostication in patients with acute symptomatic pulmonary embolism. Arch. Intern. Med..

[7] Park K.C., Gaze D.C., Collinson P.O., Marber M.S. (2017). Cardiac troponins: From myocardial infarction to chronic disease. Cardiovasc. Res..

[8] Konstantinides S., Geibel A., Olschewski M., Kasper W., Hruska N., Jäckle S., Binder L. (2002). Importance of cardiac troponins I and T in risk stratification of patients with acute pulmonary embolism. Circulation.

[9] Panteghini M., Pagani F., Bonetti G. (1999). The sensitivity of cardiac markers: An evidence-based approach. Clin. Chem. Lab. Med..

[10] Meyer T., Binder L., Hruska N., Luthe H., Buchwald A.B. (2000). Cardiac troponin I elevation in acute pulmonary embolism is associated with right ventricular dysfunction. J. Am. Coll. Cardiol..

[11] Kline J.A., Hernandez-Nino J., Rose G.A., Norton H.J., Camargo C.A. (2006). Surrogate markers for adverse outcomes in normotensive patients with pulmonary embolism. Crit. Care Med..

[12] Jiménez D., Díaz G., Molina J., Martí D., Del Rey J., García-Rull S., Escobar C., Vidal R., Sueiro A., Yusen R.D. (2008). Troponin I and risk stratification of patients with acute nonmassive pulmonary embolism. Eur. Respir. J..

[13] Douketis J.D., Leeuwenkamp O., Grobara P., Johnston M., Söhne M., Ten Wolde M., Büller H. (2005). The incidence and prognostic significance of elevated cardiac troponins in patients with submassive pulmonary embolism. J. Thromb. Haemost..

[14] Jiménez D., Uresandi F., Otero R., Lobo J.L., Monreal M., Martí D., Zamora J., Muriel A., Aujesky D., Yusen R.D. (2009). Troponin-based risk stratification of patients with acute nonmassive pulmonary embolism: Systematic review and metaanalysis. Chest.

[15] Lankeit M., Friesen D., Aschoff J., Dellas C., Hasenfuß G., Katus H., Konstantinides S., Giannitsis E. (2010). Highly sensitive troponin T assay in normotensive patients with acute pulmonary embolism. Eur. Heart J..

[16] Lankeit M., Jiménez D., Kostrubiec M., Dellas C., Hasenfuss G., Pruszczyk P., Konstantinides S. (2011). Predictive value of the high-sensitivity troponin T assay and the simplified pulmonary embolism severity index in hemodynamically stable patients with acute pulmonary embolism: A prospective validation study. Circulation.

[17] Becattini C., Vedovati M.C., Agnelli G. (2007). Prognostic value of troponins in acute pulmonary embolism: A meta-analysis. Circulation.

[18] Cowie M.R., Mendez G.F. (2002). BNP and congestive heart failure. Prog. Cardiovasc. Dis..

[19] Clerico A., Iervasi G., Mariani G. (1999). Pathophysiologic relevance of measuring the plasma levels of cardiac natriuretic peptide hormones in humans. Horm. Metab. Res..

[20] Martinez-Rumayor A., Richards A.M., Burnett J.C., Januzzi J.L. (2008). Biology of the natriuretic peptides. Am. J. Cardiol..

[21] Pruszczyk P., Kostrubiec M., Bochowicz A., Styczynski G., Szulc M., Kurzyna M., Fijalkowska A., Kuch-Wocial A., Chlewicka I., Torbicki A. (2003). N-terminal pro-brain natriuretic peptide in patients with acute pulmonary embolism. Eur. Respir. J..

[22] Klok F.A., Mos I.C.M., Huisman M.V. (2008). Brain-type natriuretic peptide levels in the prediction of adverse outcome in patients with pulmonary embolism: A systematic review and meta-analysis. Am. J. Respir. Crit. Care Med..

[23] Klok F.A., Van Der Bijl N., Eikenboom H.C.J., Van Rooden C.J., De Roos A., Kroft L.J.M., Huisman M.V. (2010). Comparison of CT assessed right ventricular size and cardiac biomarkers for predicting short-term clinical outcome in normotensive patients suspected of having acute pulmonary embolism. J. Thromb. Haemost..

[24] Hellenkamp K., Schwung J., Rossmann H., Kaeberich A., Wachter R., Hasenfuß G., Konstantinides S., Lankeit M. (2015). Risk stratification of normotensive pulmonary embolism: Prognostic impact of copeptin. Eur. Respir. J..

[25] Hellenkamp K., Pruszczyk P., Jiménez D., Wyzgał A., Barrios D., Ciurzyñski M., Morillo R., Hobohm L., Keller K., Kurnicka K. (2018). Prognostic impact of copeptin in pulmonary embolism: A multicentre validation study. Eur. Respir. J..

[26] Dellas C., Tschepe M., Seeber V., Zwiener I., Kuhnert K., Schäfer K., Hasenfuß G., Konstantinides S., Lankeit M. (2014). A novel H-FABP assay and a fast prognostic score for risk assessment of normotensive pulmonary embolism. Thromb. Haemost..

[27] Bajaj A., Rathor P., Sehgal V., Shetty A., Kabak B., Hosur S. (2015). Risk stratification in acute pulmonary embolism with heart-type fatty acid-binding protein: A meta-analysis. J. Crit. Care.

[28] Puls M., Dellas C., Lankeit M., Olschewski M., Binder L., Geibel A., Reiner C., Schäfer K., Hasenfuss G., Konstantinides S. (2007). Heart-type fatty acid-binding protein permits early risk stratification of pulmonary embolism. Eur. Heart J..

[29] Ye X.D., He Y., Wang S., Wong G.T., Irwin M.G., Xia Z. (2018). Heart-type fatty acid binding protein (H-FABP) as a biomarker for acute myocardial injury and long-term post-ischemic prognosis. Acta Pharmacol. Sin..

[30] Geissenberger F., Schwarz F., Probst M., Haberl S., Gruetzner S., Kroencke T., von Scheidt W., Berghaus T.M. (2019). D-Dimer Predicts Disease Severity but Not Long-Term Prognosis in Acute Pulmonary Embolism. Clin. Appl. Thromb. Hemost..

[31] Becattini C., Maraziti G., Vinson D.R., Ng A.C.C., den Exter P.L., Côté B., Vanni S., Doukky R., Khemasuwan D., Weekes A.J. (2021). Right ventricle assessment in patients with pulmonary embolism at low risk for death based on clinical models: An individual patient data meta-analysis. Eur. Heart J..

[32] Bertoletti L., Montani D., Humbert M. (2021). Right ventricle dysfunction in patients with acute pulmonary embolism supposedly at low risk for death: When evidence-based medicine rescues clinical practice. Eur. Heart J..

[33] Kaeberich A., Seeber V., Jiménez D., Kostrubiec M., Dellas C., Hasenfuß G., Giannitsis E., Pruszczyk P., Konstantinides S., Lankeit M. (2015). Age-adjusted high-sensitivity troponin T cut-off value for risk stratification of pulmonary embolism. Eur. Respir. J..

[34] Ferrari E., Moceri P., Crouzet C., Doyen D., Cerboni P. (2012). Timing of troponin I measurement in pulmonary embolism. Heart.

[35] Mismetti P., Laporte S., Pellerin O., Ennezat P.-V., Couturaud F., Elias A., Falvo N., Meneveau N., Quere I., Roy P.-M. (2015). Effect of a Retrievable Inferior Vena Cava Filter Plus Anticoagulation vs. Anticoagulation Alone on Risk of Recurrent Pulmonary Embolism. JAMA.

[36] Meyer G., Vicaut E., Danays T., Agnelli G., Becattini C., Beyer-Westendorf J., Bluhmki E., Bouvaist H., Brenner B., Couturaud F. (2014). Fibrinolysis for patients with intermediate-risk pulmonary embolism. N. Engl. J. Med..

[37] Lankeit M., Jiménez D., Kostrubiec M., Dellas C., Kuhnert K., Hasenfuß G., Pruszczyk P., Konstantinides S. (2014). Validation of N-terminal pro-brain natriuretic peptide cut-off values for risk stratification of pulmonary embolism. Eur. Respir. J..

[38] Klok F.A., Ageno W., Ay C., Bäck M., Barco S., Bertoletti L., Becattini C., Carlsen J., Delcroix M., van Es N. (2021). Optimal follow-up after acute pulmonary embolism: A position paper of the European Society of Cardiology Working Group on Pulmonary Circulation and Right Ventricular Function, in collaboration with the European Society of Cardiology Working Group on Ather. Eur. Heart J..

